# 

**Published:** 1879-09

**Authors:** 


### CONTENTS:

Article I.—Inflammation. By Wm. H. Atkinson, M. D., D. D. S. .......... 193
Article II.—Effects of Arsenic upon the Pulp By Louis Ottofy, D. D. S. .......... 202
Article III.—American Nervousness—Its Philosophy and Treatment. By Geo. M. Beard, M. D .......... 204
Article IV.—Family Taints. By Samuel Peters, M. D .......... 224

### EDITORIAL, ETC.

More Dental Schools .......... 234
Hygrometric Properties of Glycerine .......... 236
Hand-work, Head-work, and Symmetry of Brains .......... 236

### MONTHLY SUMMARY.

Remedies in Headache .......... 236

Teeth Set on Edge .......... 238

Sure Antidote to Poison .......... 238

Diseases of the Nervous System .......... 239

Method of Removing Hairs .......... 239

Neuralgia .......... 240

Virchow on the Germ Theory .......... 240

Chloral as a Counter-Irritant .......... 240

